# Machine Learning Improves Risk Stratification After Acute Coronary Syndrome

**DOI:** 10.1038/s41598-017-12951-x

**Published:** 2017-10-04

**Authors:** Paul D. Myers, Benjamin M. Scirica, Collin M. Stultz

**Affiliations:** 1 Department of Electrical Engineering and Computer Science, Massachusetts Institute of Technology (MIT), Cambridge, MA 02139 USA; 2 TIMI Study Group, Cardiovascular Division, Department of Medicine, Brigham and Women’s Hospital and Harvard Medical School, Boston, MA 02115 USA; 3 Institute for Medical Engineering and Science, MIT, Cambridge, MA 02139 USA

## Abstract

The accurate assessment of a patient’s risk of adverse events remains a mainstay of clinical care. Commonly used risk metrics have been based on logistic regression models that incorporate aspects of the medical history, presenting signs and symptoms, and lab values. More sophisticated methods, such as Artificial Neural Networks (ANN), form an attractive platform to build risk metrics because they can easily incorporate disparate pieces of data, yielding classifiers with improved performance. Using two cohorts consisting of patients admitted with a non-ST-segment elevation acute coronary syndrome, we constructed an ANN that identifies patients at high risk of cardiovascular death (CVD). The ANN was trained and tested using patient subsets derived from a cohort containing 4395 patients (Area Under the Curve (AUC) 0.743) and validated on an independent holdout set containing 861 patients (AUC 0.767). The ANN 1-year Hazard Ratio for CVD was 3.72 (95% confidence interval 1.04–14.3) after adjusting for the TIMI Risk Score, left ventricular ejection fraction, and B-type natriuretic peptide. A unique feature of our approach is that it captures small changes in the ST segment over time that cannot be detected by visual inspection. These findings highlight the important role that ANNs can play in risk stratification.

## Introduction

The term acute coronary syndrome (ACS) refers to a spectrum of conditions that are primarily attributable to inadequate blood flow to the myocardium following an acute cholesterol plaque rupture or erosion leading to thrombus formation^1^. In 2010, more than 1.1 million unique hospitalisations were ascribed to ACS and it is estimated that in the United States more than 780,000 people will experience an ACS each year^1,2^.

The effective treatment of patients who have suffered an ACS necessitates an accurate assessment of that patient’s risk of subsequent adverse cardiovascular events, thereby permitting accurate risk stratification and the delivery of appropriate therapy. Invasive procedures, for example, are inappropriate for patients whose risk of future adverse events is lower than the risk of the procedure itself. Patients who are deemed to be very high risk benefit from aggressive, higher risk strategies and closer follow-up^3,4^. Therefore, the process of categorising patients according to their risk – a process called risk stratification – is a core part of the management of patients post-ACS.

Early risk-stratification plays an especially important role in the management of patients after an ACS. Patients who are identified as high risk using traditional risk metrics benefit from invasive therapies (such as percutaneous coronary intervention) within 24–48 hours of presentation^5,6^. Risk scores such as the Thrombolysis in Myocardial Infarction (TIMI) and the Global Registry of Acute Coronary Events (GRACE) scores are widely used tools that are calculated using a patient’s presenting signs and symptoms, historical data - information that is available at the time of presentation – and the results of laboratory studies that can be obtained within minutes to hours after presentation^7–9^.

Patients who present with ACS are typically divided into two categories – those with ST segment elevation on their presenting electrocardiogram (STE-ACS) and those with non-ST segment elevation (NSTE-ACS). Risk assessment in patients with NSTE-ACS can be problematic because although their short-term in-hospital mortality is lower relative to patients who present with STE-ACS, they have similar rates of CVD in the long term^10–13^. Traditional metrics that assess the risk of future adverse cardiovascular events after NSTE-ACS use a range of clinical variables to estimate the patient risk^7,9^. Despite this, an accurate assessment of patient risk remains a difficult task. Many risk scores, for example, fail to capture a significant number of deaths in certain patient cohorts^14,15^.

Given the importance of the ST segment in identifying high-risk patient subgroups, the presence and extent of ST-segment deviation are often components in patient risk models^7,12,16^. However, while physicians are well trained to assess the extent of ST segment deviation by eye, subtle ST segment deviations that cannot be detected by eye may also carry important prognostic information. These considerations motivate the development of more sophisticated analysis techniques that exploit the detection of subtle ST segment changes that may be unrecognized by visual inspection alone.

Herein we present a method for combining features from the patient record and the ECG to predict cardiovascular death (CVD) within one year after a NSTE-ACS. A key feature of the method is that it not only utilizes traditional patient demographic and medical history information, but it also takes advantage of the continuous ECG waveform that is typically available from standard Holter monitoring. Unlike other risk assessment tools that only use a single feature of the surface ECG on presentation, such as the level of ST-segment depression, our method applies an Artificial Neural Networks (ANN) to the precise time series of ST segment changes after admission with a NSTE-ACS. The prognostic ability of the final model is evaluated on two independent datasets composed of post-ACS patients. Using an ANN that is trained on a small amount of Holter data – we arrive at a model that outperforms traditional risk models and other risk metrics that use considerably more data.

## Results

### Patient Characteristics

The study populations consisted of two patient cohorts (Table 1) that were derived from two different clinical studies used in previous work to evaluate the performance of several computational biomarkers^14,17,18^. The first study^19,20^ included 6,560 patients with interpretable continuous ECG data, and the second study^21^ included 990 patients with interpretable ECG data. All patients in both cohorts were enrolled, and ECG collection began, within 48 hours after presenting with symptoms consistent with a NSTE-ACS. From these datasets, we restricted our analysis to patients who had at least one day of continuous ECG signal and values for seven baseline characteristics: age, gender, current smoker, history of hypertension, history of diabetes, previous myocardial infarction (MI), and a history of previous angiography. These features correspond to the subset of features that were in common to both cohorts. Using these criteria, 4,395 patients from Cohort-1 and 861 patients from Cohort-2 were used in the analysis.

**Table 1:** Baseline patient characteristics for Cohort-1 and Cohort-2. IQR is interquartile range; MI is myocardial infarction; LVEF is left ventricular ejection fraction; BNP is brain natriuretic peptide.

	Cohort-1	Cohort-2
Population Size	4395	861
Cardiovascular Deaths	149 (3.39%)	14 (1.63%)
Age in Years, Median (IQR)	63 (55 to 71)	63 (54 to 72)
Female, %	35	36
Diabetes Mellitus, %	33	24
Hypertension, %	73	69
Current Smoker, %	26	56
Previous MI, %	33	26
Previous Angiography, %	34	60
TIMI Risk Score, %		
Low (1 to 2)	27	—
Moderate (3 to 4)	53	—
High (5 to 7)	20	—
LVEF		
≤40%, %	9	—
>40%, %	58	—
BNP		
>80 pg/ml, %	29	—
≤80 pg/ml, %	41	—

### Logistic Regression Models with ST-segment Based Features

**Figure 1:**
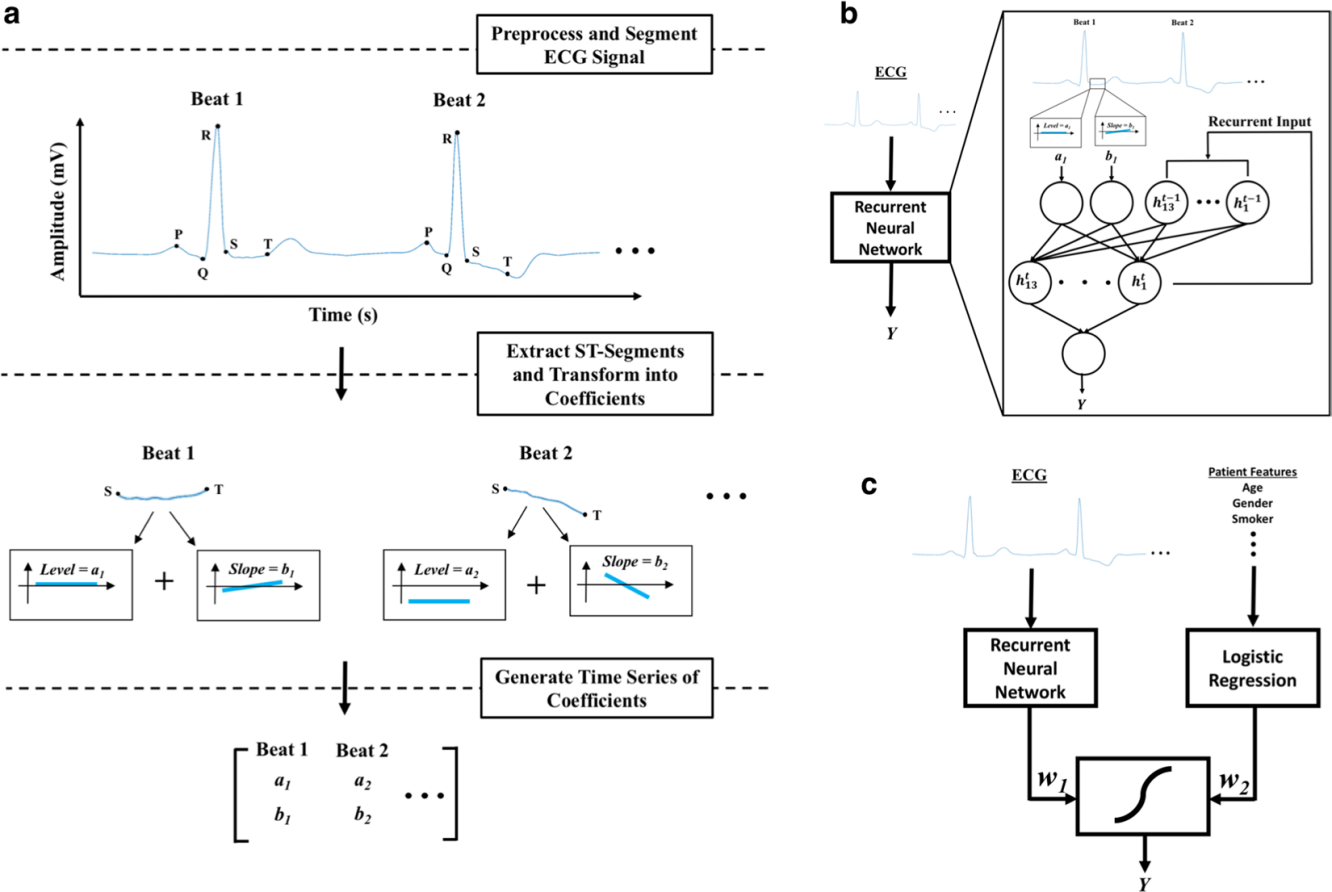
(**a**) ST segment feature extraction process. The ECG signal is first preprocessed to remove noise and identify clean beats, then segmented to delineate the various waveforms comprising each beat. The ST segments are then isolated and a mathematical transformation is applied to extract the coefficients describing the morphology of the segment, which are then applied as input to the model. (**b**) Schematic representation of the recurrent neural network (RNN) model. The raw ECG signal is preprocessed and the first two Legendre polynomial coefficients are extracted for each beat, as shown in (**a**). The hidden units are denoted by ***h***_***i***_^***t***^ and the recurrent inputs are denoted by ***h***_***i***_^***t-1***^, where *t* is the current time step and *t-1* is the previous time step. ***Y*** is the prediction. (**c**) Schematic representation of the ANN model. The predictions from the RNN and LR models are multiplied by weights ***w***_***1***_ and ***w***_***2***_, and then input to a final neuron with sigmoidal activation function, yielding prediction ***Y***.

Recently, a method for mathematically representing the morphology of the ST segment was proposed to distinguish between ischemic and non-ischemic ST segment events in an ECG tracing^22^. In this approach, the ECG signal is first segmented into beats and the ST segment is then identified and extracted from each beat. A mathematical transformation based on Legendre polynomials is then applied to the ST segments, yielding a set of coefficients that capture clinically relevant morphological features. The first coefficient, for example, represents the level relative to the isoelectric line – thereby quantifying the extent of ST-segment deviation – and the second coefficient represents the slope of the ST segment. All higher-order coefficients describe the ST segment’s curvature. The ECG signal of each patient can therefore be expanded into a sequence of these coefficients, with each point in the sequence corresponding to a beat in the original ECG signal (Fig. 1a). An attractive feature of this approach is that the representation of the ST segment in terms of Legendre coefficients can capture small changes in the ST segment that fall below the resolution of the human eye.

To test the discriminatory value of this approach we incorporated features derived from this representation of the ST-segment into logistic regression (LR) models designed to predict CVD one year after a NSTE-ACS. Three models were tested: LR_Hx_, LR_ST_, and LR_Hx+ST_.

The LR_Hx_ model only uses information from the patient history and demographic data. The seven features used were age, gender, whether the patient was a smoker at the time of enrollment, history of hypertension, history of diabetes, previous myocardial infarction (MI), and previous angiography; i.e., features that were in common to both patient cohorts.

The LR_ST_ model uses four features corresponding to the mean and standard deviation of the first two coefficients representing the level and slope of the ST segments of the first 50 beats from the Holter – a time scale corresponding to less than one minute in this patient population. Parenthetically, as we exclude regions of the Holter signal that have a low signal to noise ratio, and early portions of the Holter recordings in this dataset often contain significant noise, we sometimes require more than one minute of data to obtain 50 usable beats (see Materials and Methods).

The LR_Hx+ST_ logistic regression model uses the seven features in the LR_Hx_ model plus the four ST-segment based features in the LR_ST_ model.

All three models were applied to the Cohort-1 dataset using a bootstrapping procedure in which the entire Cohort-1 was randomly partitioned into a training set containing 80% of the data and a testing set containing 20% of the data, while ensuring that each training and test set had the same percentage of CVDs (~3.4%). This random partitioning was repeated 1000 times. For each partition, the three models were trained on the training sets (80% of the data) and then applied to the testing set (20% of the data). Results are reported as averages over the 1000 testing sets.

Of the three models considered, the LR_Hx+ST_ model had the greatest discriminatory power (Table 2). The small improvement in the AUC of the LR_ST_ model over the LR_Hx_ model was statistically significant with *p* < 0.05, and the improvement in the AUC of the LR_Hx+ST_ over the LR_Hx_ and LR_ST_ models was also significant, with *p* < 0.001 in both cases.

**Table 2:** Performance of different classification models.represent averages over 1,000 trials.

**Model**	**AUC**	**95% CI**
LR_Hx_	0.695	0.581–0.809
LR_ST_	0.701^*^	0.587–0.814
LR_Hx+ST_	0.734^**^	0.623–0.845
LR_Hx+MV_	0.727	0.615–0.839
LR_Hx+HRV_	0.720	0.607–0.832
LR_Hx+DC_	0.705	0.591–0.818
TRS	0.670	0.555–0.786
RNN	0.689	0.575–0.803
ANN	0.743^***^	0.633–0.853
**Metric**	**Mean **	**95% CI**
NRI of ANN w.r.t. TRS	0.065	0.059–0.071
Patients Correctly Reclassified	87	86.4–87.6

AUCs of different models (and the TIMI NSTE-ACS risk score) using the Cohort-1 dataset and 2 Category NRI of the best performing model relative to the TIMI Risk Score (TRS). AUC is area under the curve; CI is confidence interval; MV is morphologic variability, HRV is heart rate variability LF/HF (see Materials and Methods); DC is deceleration capacity; TRS is the TIMI NSTE-ACS Risk Score; RNN is recurrent neural network. AUCs represent averages over 1,000 trials. NRI is two-category net reclassification index. Values *Improvement over LR_Hx_ significant with *p* < 0.05. ^**^Improvement over LR_Hx_, LR_ST_, LR_Hx+MV_, LR_Hx+HRV_, and LR_Hx+DC_ significant with *p* < 0.001. ^***^Improvement over all other models significant with *p* < 0.001.

For comparison, we evaluated the performance of three other logistic regression models that incorporate ECG information in various forms. In a previous work, we evaluated the performance of several ECG-based metrics for risk stratification post-NSTE-ACS using the Cohort-1 dataset^18^. Morphologic Variability (MV), Heart Rate Variability (HRV), and Deceleration Capacity (DC) are three ECG-based metrics that have been associated with adverse outcomes in patients with cardiovascular disease^17,18,23,24^. We therefore constructed three additional logistic regression models using these metrics. Each model combined all seven patient features with one of these metrics: i.e., LR_Hx+MV_, LR_Hx+HRV_, and LR_Hx+DC_. It is important to note that each of these ECG metrics use at least 24 hours’ worth of ECG data while the LR_ST_ and LR_Hx+ST_ models only use 50 beats from the Holter monitor.

The LR_Hx+MV_ model offered the highest AUC of all the comparison models tested (Table 2), followed by the LR_Hx+HRV_, LR_Hx+DC_ models; however, all three AUCs are below that of the LR_Hx+ST_ model (*p* < 0.001). Lastly, the AUC associated with the TIMI Risk Score for NSTE-ACS (TRS)^7^, which includes information about the presence of 1 mm ST segment depression on presentation, falls below all of the logistic regression models (Table 2).

### An Artificial Neural Network with ST-segment Based Features

These results suggest that the ST-segment based features derived from an automated analysis of the ST-segment have discriminative power. However, since the logistic regression models do not exploit the fact that we have a time-series of ST-segments, we developed a neural network model that could effectively use these data. In particular, recurrent neural networks (RNNs) – a type of ANN – provide a formalism for modeling time series data that we exploit in this work^25,26^. The RNN architecture we use is shown in Fig. 1b. In addition, since our data suggest that combining features derived from the patient history with ST-segment based features leads to improved performance, we also evaluated a model that combines the output from a RNN and the LR_Hx_ models into one model (ANN, Fig. 1c).

The ANN model offering a markedly improved AUC over the RNN model (*p* < 0.001) and the best discriminatory value of all models tested (*p* < 0.001 for all pairwise comparisons) (Table 2). The category-free net reclassification index (NRI)^27^ of ANN relative to the LR_Hx+ST_ model was 0.18 (18%), albeit the standard deviation of this value over 1,000 bootstrapped datasets was 0.451. The two-category NRI with respect to the TIMI risk score was 0.065 (6.5%); over the 1000 bootstrap trials, an average of 87 patients were correctly reclassified using the ANN model with a standard deviation of 9.4 patients (Table 2).

### Univariate Association of Models with CVD in Cohort-1

We assessed the univariate association of the LR_Hx+ST_ and ANN models with CVD at one year and 60, 30, and 14 days by calculating Hazard Ratios (HRs) using Cox proportional hazard models (highest vs. other quartiles, Table 3). For comparison, we included results for the TIMI NSTE-ACS risk score. HRs were deemed statistically significant if the lower 95% confidence interval remained above one. All HRs for the LR_Hx+ST_ and ANN models were statistically significant with the LR_Hx+ST_ model showing the highest association with CVD. The TIMI risk score had significant HRs for CVD at one year, 60 days, and 14 days, although not at 30 days. One-year, 30-day and 14-day HRs for the LR_Hx+ST_ model were significantly higher than those associated with the TIMI risk score (*p* < 0.05), and one-year, 60-day, and 30-day HRs for the ANN model were consistently higher than the TIMI risk score (*p* < 0.05).

**Table 3:** Univariate Hazard Ratios (highest vs. other quartiles) calculated from the Cohort-1 dataset. CI is confidence interval.

	Hazard Ratio	95% CI
LR_Hx+ST_		
1-Year	4.744	2.232–10.100
60-Day	5.918	1.841–19.892
30-Day	6.607	1.583–30.793
14-Day	6.606	1.229–39.907
ANN		
1-Year	4.510	2.126–9.584
60-Day	4.943	1.580–16.202
30-Day	5.260	1.342–22.544
14-Day	5.546	1.068–32.413
TRS		
1-Year	3.667	1.761–7.638
60-Day	3.822	1.298–11.428
30-Day	3.309	0.920–12.512
14-Day	5.417	1.133–28.885

Hazard Ratios and CIs represent averages over 1,000 trials (each trial yields one HR and one 95% CI).

### Multivariate Association of Models with CVD in Cohort-1

The HRs for the LR_Hx+ST_ and ANN models for CVD at one year were adjusted for the TRS, left ventricular ejection fraction (LVEF), and brain natriuretic peptide (BNP), all of which are used clinically to identify high-risk patients^28–31^; the NSTE-ACS TRS model was also adjusted for LVEF and BNP only (Table 4). For the multivariate HRs using the TRS, LVEF, and BNP, patients were grouped in the high-risk category using the following criteria: TRS > 4, LVEF ≤ 40%, and BNP > 80 pg/mL.

**Table 4:** Multivariate Hazard Ratios on Cohort-1.

	**1-Year HR**	**95% CI**
LR_Hx+ST_		
TRS	3.624	1.598–8.232
TRS + LVEF	2.823	1.016–7.924
TRS + BNP	3.430	1.243–9.565
TRS + LVEF + BNP	3.564	0.979–13.821
ANN		
TRS	3.473	1.555–7.771
TRS + LVEF	2.956	1.075–8.221
TRS + BNP	3.431	1.267–9.419
TRS + LVEF + BNP	3.722	1.035–14.339
TRS		
LVEF	3.085	1.214–7.880
BNP	3.405	1.400–8.312
LVEF + BNP	3.118	1.001–9.875

HR is hazard ratio (highest vs. other quartiles); CI is confidence interval; LR is logistic regression; TRS is TIMI risk score; LVEF is left ventricular ejection fraction; BNP is brain natriuretic peptide. HRs and CIs represent averages over 1,000 trials (each trial yields one HR and one 95% CI).

The ANN model had the highest HR for the TRS + LVEF, TRS + BNP, and TRS + LVEF + BNP adjustments, while the LR_Hx+ST_ model had the highest HR for the TRS adjustment. More importantly, the HR for the ANN model remains statistically significant at one year after accounting for all covariates and is larger than the HR for the TIMI risk score (*p* < 0.05).

### Univariate Association of Models with CVD in Specific Patient Populations

**Figure 2:**
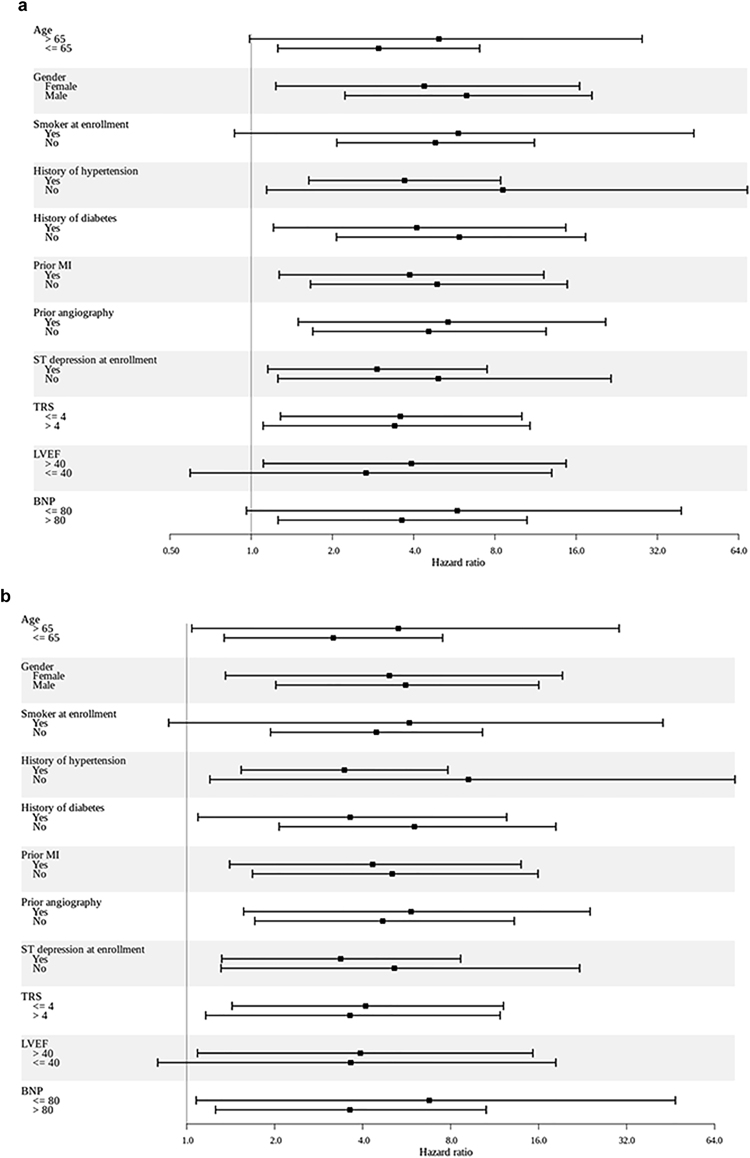
Univariate hazard ratios (highest vs. other quartiles) in various subpopulations of Cohort-1 for the (**a**) LR_Hx+ST_ and (**b**) ANN models. The black squares show mean values, and the horizontal bars represent 95% confidence intervals (CIs). The number of deaths in each subgroup is given in Table S4. Hazard ratios and CIs represent averages over 1,000 trials (each trial yields one HR and one 95% CI).

We further evaluated the performance of the LR_Hx−ST_ and ANN (Fig. 2) models on specific patient populations. Univariate HRs for both models were statistically significant in a number of subgroups that are considered to be low-to-moderate-risk using traditional metrics; e.g., patients with a TIMI risk score $\le$ 4, LVEF > 40%, no history of diabetes, prior angiography, or a prior history of myocardial infarction. Most notably, the one-year HR for LR_Hx−ST_ and ANN are significant in patients who do not have at least 1 mm ST segment depression at enrollment. Additionally, the ANN model, unlike LR_Hx−ST_ model, has statistically significant univariate HRs for patients who have BNP < 80 pg/ml, and for patients < 65 years of age.

### Association of Models with CVD in Cohort-2

**Figure 3:**
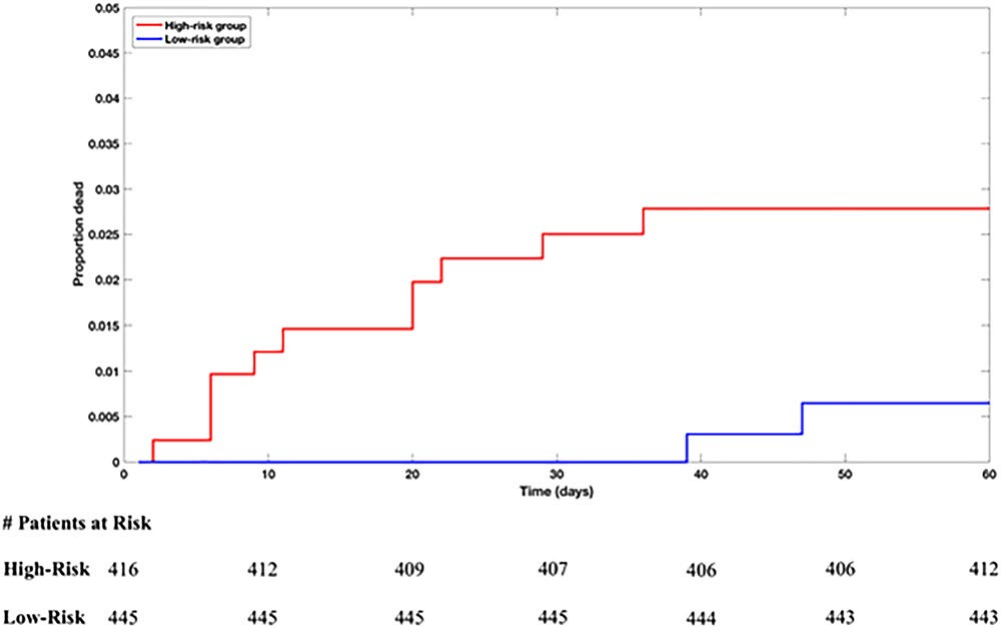
Kaplan-Meier survival curves for the ANN model on Cohort-2 (red is the high-risk subgroup and blue is the low-risk subgroup). The cutoff value used to define the high-risk subgroup was chosen based on results on Cohort-1. The number of patients remaining at each labelled time point in each risk group is shown below the plot.

When applying the models to Cohort-2 the LR_Hx−ST_ and ANN models were trained on the entire Cohort-1 dataset and then applied to the entire Cohort-2 dataset. In this sense, the Cohort-2 dataset was used as an independent holdout set to evaluate the ability of the models developed on Cohort-1 to generalise to unseen data. The ANN model achieved an AUC of 0.767 with a 95% confidence interval of 0.615–0.918 on Cohort-2, as opposed to an AUC of 0.758 with a 95% confidence interval of 0.605–0.911 for the LR_Hx+ST_ model. Univariate one-year, 60-day, 30-day, and 14-day HRs were calculated for both models, and the ANN model was found to have statistically significant results for all time periods considered; by contrast, the LR_Hx+ST_ model only had statistically significant results for one-year and 30-day outcomes (Table 5). HRs for Cohort-2 were calculated using the upper-quartile of the raw predictions from Cohort-1. Patients in Cohort-2 were deemed high-risk if their raw prediction value for a given classifier exceeded the upper-quartile cutoff derived from Cohort-1. Because the cutoff value was derived from Cohort-1, it was tailored to that particular dataset. Indeed, the Cohort-1 cutoff value is a double precision number with 16 significant digits. To improve the generalisability to other datasets, the upper-quartile cutoff derived from Cohort-1 was truncated to one significant digit. Multivariate HRs were not computed, as TRS, LVEF, and BNP measurements were not available in the Cohort-2 dataset. Kaplan-Meier survivor curves were generated the ANN (Fig. 3) and LR_Hx+ST_ (Supplementary Fig. S3) models for a follow-up period of 60 days.

**Table 5:** Univariate Hazard Ratios (highest vs. other quartiles) for Cohort-2. CI is confidence interval; LR is logistic regression.

	**Hazard Ratio**	**95% CI**
LR_Hx+ST_		
1-Year	3.661	1.269–10.559
60-Day	2.992	0.979–9.146
30-Day	4.764	1.379–16.457
14-Day	4.750	0.959–23.534
ANN		
1-Year	4.420	1.232–15.853
60-Day	4.773	1.313–17.349
30-Day	6.885	1.780–26.630
14-Day	15.711	3.170–77.855

## Discussion

Our results demonstrate that signal processing and machine learning can be used to generate patient risk models with improved performance compared to traditional logistic regression techniques. While machine-learning techniques have been successfully applied in a number of domains of clinical medicine, applications in cardiovascular risk stratification have been somewhat limited. Several studies have attempted to apply machine learning models based on ANNs to a number of clinical problems, including the diagnosis of myocardial infarction^32^, risk stratification after myocardial infarction/unstable angina^33,34^ and ACS^35^ but have found ANNs to offer few, if any, advantages over more conventional techniques based on logistic regression and Bayesian classification. What is lacking in these studies, however, is an attempt to use machine learning techniques to exploit aspects of clinical data that cannot be approached by conventional techniques, as the same sets of variables were used in both the conventional and ANN models.

In our study population, the TIMI NSTE-ACS risk score has an AUC of 0.67 and therefore remains a useful discriminatory metric that can identify patients at elevated risk of death post NSTE-ACS. However, only 20% of the patients in the Cohort-1 study belonged to the highest TRS category, and 54% of the deaths in Cohort-1 dataset occurred in patients in the low- and moderate-risk categories. Hence, the TRS by itself is insufficient to accurately risk-stratify the majority of patients in this population.

Comparing the relative performance of the first three models (Table 2) demonstrates both the predictive power of the feature set derived from the ECG, as well as the utility of combining these features with those from the patient record. The LR_ST_ model uses only mean and standard deviation values derived from the first 50 usable beats of the ECG record for each patient, but offers a statistically significant improvement in performance over the LR_Hx_ model, which only uses patient features that are strongly associated with adverse cardiovascular outcomes. While both models perform comparably individually, the best discriminatory power is achieved by combining these two types of clinical data into the LR_Hx+ST_ model.

Further gains in performance can be achieved by replacing the simple summary statistics for the ECG, which we use in the LR_Hx+ST_ model, with a RNN that is trained on the ECG time series alone. Unlike some neural network approaches that construct models that contain many layers and thousands of free parameters^26,36^, a driving force behind the design of our RNN was to minimise the number of free parameters relative to the number of patients in our dataset. This strategy was used to minimise overfitting of the model and thereby help ensure that the model will generalise to unseen data. Indeed, our RNN has only one hidden layer and the ANN model has a total of 217 free parameters. By contrast, each bootstrapping trial used a training set that contained 3,516 patients and the final model, which was applied to Cohort-2, used the entire Cohort-1 dataset for training (4,395 patients). Additionally, concerns about overfitting were further mitigated by using a regularisation procedure for the ANN model as described in the Materials and Methods section.

Of all models considered, the ANN model achieves the highest AUC and therefore the best discriminative power. Moreover, the ANN model retains its predictive ability in a number of subgroups that are not typically considered to form a high-risk population. Univariate hazard ratios for the ANN are significant in patients who do not present with ST-segment depression, patients who have a TIMI Risk Score that is less than or equal to 4, patients who have an LVEF > 40, and patients who have a BNP $\le$ 80 pg/ml. These results are encouraging because most deaths in Cohort-1 occur in patients who do not fall into these high-risk categories. Methods that can find high-risk patients, who would not be identified as such using traditional metrics, are therefore of particular interest.

The ANN model is the only model that exhibits statistically significant HRs when adjusted for all combinations of the TRS, LVEF, and BNP, and it also has statistically significant HRs at one year, 60 days, 30 days, and 14 days when applied to an independent dataset, while the LR_Hx+ST_ model does not. The ANN model utilizes the raw time series information while the LR_Hx+ST_ model uses simple summary statistics derived from this same time series, thereby losing this temporal information. Thus, the added predictive power of the model appears to come from the ability of the RNN to take into account the evolution of the ST segment morphology over time.

The ANN model uses a relatively small amount of data – the first 50 beats in the ECG recording – to achieve this improved performance. It should be stressed, however, that while the ST segment morphology features were derived from 50 beats, corresponding to less than one minute of data in this patient population, we generally require more time to secure 50 beats that have ST segments that can be clearly identified using existing automated methods. Since ECG pre-processing involves removing 5-minute segments that have low signal to noise ratios, regions near the beginning of noisy Holter tracings are deleted. (Parenthetically, segmenting the ECG signal into 5-minute segments was done to be consistent with the preprocessing procedure used for the calculation of other ECG-based metrics including HRV and MV^17,37^). Overall, approximately 45 minutes of data were required on average to obtain the requisite number of beats where the beginning and ending of each ST segment could be unambiguously identified using an automated method for identifying individual components of each beat^38^.

Of note, existing risk metrics, like the TIMI and GRACE scores, require the results of lab values that are generally available within approximately one hour after the patient presents with signs and symptoms of an ACS. Consequently, in the setting of noisy Holter data, all of the information needed to estimate patient risk with the ANN model can be obtained in a similar time frame as the information needed to use traditional risk scores; furthermore, if the data are low-noise, all of the information needed to produce a risk estimate with the ANN model can be obtained within minutes, allowing clinicians to obtain estimates on shorter time scales than with traditional metrics. Risk assessment within the first few hours after presentation plays an important role in the management of ACS as patients who are identified as high risk may benefit from invasive strategies within 24–48 hours after the diagnosis of ACS is made^6^. Therefore, we focused on methods that would use a small amount of Holter data, thereby ensuring that we would arrive at a model that could be used to quickly assess patient risk. Interestingly, although our method uses a relatively small amount of ECG data, it has greater discriminatory power relative to other ECG-based methods such as MV^17^, HRV^39^, and DC^24^, that require at least 24 hours of data.

One limitation of this study is that a single sampling rate of 128 Hz was used for collecting the ECG data in both cohorts; thus, no claims can be made about the performance of the model on data sampled at different rates. However, only the first two Legendre polynomial coefficients, which represent the level and slope of the ST-segment, were used in the analysis, and it is unlikely that changing the sampling rate will drastically change the mean value of the ST segment or its slope. A second limitation of this study is that an automated algorithm was used to extract the ST-segments from the ECG data, so the criteria used by the algorithm to identify the endpoints of the ST-segment may influence the final values of the coefficients obtained. Nonetheless, if the ECG data are relatively free of noise, small changes in the ST-segment delineation are not expected to significantly affect the final results.

Although the predictive accuracy of a model is an important consideration when deciding to deploy a model in a clinical setting, the interpretability of a model is also an important concern. Models that are readily interpretable are more easily accepted by the medical community and, more importantly, can lead to new hypotheses that form the basis of future clinical studies.

A useful property of logistic regression models is that the magnitudes and signs of the weights of the model relative to each other can provide some insight into the importance of the various features in determining the prediction of the model; neural networks, by contrast, lack such interpretability due to their complexity. The ANN model consists of both logistic regression and neural network components, and therefore, parts of the model are interpretable, while parts are not (see Fig. 1c). The portion of the model that takes the patient features as inputs contains conventional logistic regression weights, which may be compared with each other to derive some insight into the relative importance of the various baseline features. The weights used to combine the output of the RNN and the output of the patient feature logistic regression, denoted *w*_1_ and *w*_2_ in Fig. 1c, provide some insight into the relative importance of the ECG data, as used in the RNN, versus the patient features. Since the structure of the RNN model (Fig. 1b) prohibits an easy interpretation of the model weights, one way to develop some understanding of what the model learns is to apply various types of input sequences and observe the output. Therefore, to gain insight into the types of input ECG sequences that are associated with increased risk we input a number of different handcrafted sequences of ST segment coefficients to the RNN. These experiments suggest that random sequences, where adjacent time points are completely uncorrelated with each other, tend to have higher RNN outputs relative to more correlated sequences. These data are consistent with our prior observations that significant variability in the morphology of adjacent ECG beats are associated with an increased risk of adverse cardiovascular events post-ACS^18^. More importantly, these observations demonstrate that insights into what an ANN has “learned” can be garnered by experimenting with different input sequences that have distinct characteristics.

Finally, the ability of the ANN model to identify high-risk patients in two independent datasets argues that this model generalises well to unseen data. These observations demonstrate that machine learning provides a framework for fruitfully combining disparate pieces of data to yield models with improved performance.

## Materials and Methods

### Experimental Design

The first study included 4,557 patients with interpretable continuous ECG data^18^. Continuous monitoring was done for up to seven days following randomisation with a three-lead digital Holter monitor at a sampling rate of 128 Hz. Patients with fewer than 50 usable beats in the first day of ECG data or who were missing any of the seven patient features were not considered in the analysis; using these criteria, 4,395 of the 4,557 patients remained.

The second study included interpretable ECG data from 909 patients^17^. Continuous ECG monitoring was conducted during the first four to seven days after randomisation. As with the Cohort-1 dataset, patients with fewer than 50 clean beats in the first day of ECG data or who were missing any of the seven patient features were not considered in the analysis; using these criteria, 861 of the 909 patients remained.

Continuous-valued patient features in both cohorts, including age, MV, HRV, and DC, were normalized to fall within [0,1] by subtracting the minimum and dividing by the range of each variable.

### ECG Preprocessing

Preprocessing of the ECG signal for noise removal followed the procedure outlined in^17^. The signal was first divided into 5-minute segments. Baseline wander was removed by applying a median filter to the ECG signal, and wavelet filtering with a soft threshold was used to remove high-frequency noise in each segment. The signal was then normalized by the mean R-wave amplitude and the Physionet Signal Quality Index^40^ was used to remove noisy signal segments. Segmentation of each beat of the signal into its constituent components (P-wave, QRS-complex, ST-segment, T-wave) was done using a previously published method based on wavelet transforms^38^.

### ST-Segment Morphology Feature Extraction

For each patient, the ECG signal for the first day of continuous monitoring was preprocessed as described above. The S-wave and T-wave labels returned by the wavelet transform-based segmentation algorithm were used to delineate the ST-segment of each beat, and 16 samples were chosen around the midpoint between these two labels. Beats that were missing S-wave or T-wave labels, contained fewer than 16 samples, or contained more than 32 samples were discarded, since the ST-segments of these beats were determined to be too noisy to use. Gram-Schmidt orthonormalisation was then applied to the extracted segments to produce an intermediate time series of 16-component vectors. The standard deviation of each component of this intermediate time series was then computed, and each component of this intermediate time series was normalized by the corresponding standard deviation to produce the final time series of Legendre polynomial coefficients. Although all patients used in the training and testing of the network possessed at least one day of continuous ECG data, the number of clean beats varied markedly across patients, necessitating the use of a fixed number of beats. Due to limits in the recurrent memory of the RNN, a window of the first 50 beats, which corresponds to approximately one minute of data, was used for each patient. Patients possessing fewer than 50 clean beats were not included in the analysis. Although 16 Legendre polynomials were derived for each ST-segment, only the first two were used in subsequent analyses; this choice was made for two reasons. The principal reason is that the first two coefficients, which represent the level and slope of the ST-segment, are of primary clinical significance when diagnosing conditions that affect the morphology of the ST-segment. The second reason is that the higher-order coefficients were found to capture the noise inherent in the signal due to the low sampling rate. A detailed description of the feature extraction process is given in the Supplementary Materials.

### LR_Hx_ Classifier

Although the Cohort-1 study recorded a relatively rich set of patient features, most of these features were not available in the Cohort-2 study. Thus, the logistic regression model was trained using only those features in common between the two datasets. This restriction offers the two-fold benefit of reducing the risk of overfitting and increasing the applicability of the model to clinical situations in which only a limited amount of patient information is available. Moreover, models trained using fewer features tend to offer improved interpretability. L2-regularisation was employed to reduce overfitting and to improve the generalisability of the model; the cost parameter for the regularisation was found using three-fold cross-validation. Patients missing any of the required features were not included in the analysis.

### LR_ST_ and LR_Hx+ST_ Classifiers

The time series of the first two Legendre polynomial coefficients derived from the first usable 50 beats in the ECG signal were summarized using the mean and standard deviation to form the LR_ST_ model. The mean and standard deviation of each of the first two coefficients used in the LR_ST_ model were combined with the seven patient record features used in the LR_Hx_ model to generate the LR_Hx+ST_ model. L2 regularisation was applied to both the LR_ST_ and LR_HX+ST_ models to mitigate overfitting to the training data using three-fold cross-validation.

### RNN Classifier

The RNN (Fig. 1b), which was used to process the Legendre polynomial coefficient time series described above, consisted of an input layer of two units, a hidden layer of 13 units, and an output layer of one unit. The number of weights in the network, which is dictated largely the number of hidden units, was required to be less than 10% of the number of patients in the training set in order to avoid overfitting. Additional details about the RNN are provided in the Supplementary Materials.

### ANN Classifier

The outputs of the LR_Hx_ and RNN classifiers were combined using a second level of L2-regularized logistic regression with three-fold cross-validation (Fig. 1c). The LR_Hx_ and RNN classifiers were trained independently on a given training set, and the outputs of each classifier on this training data were used as input to a second level of logistic regression. The weights of this second level of logistic regression, denoted as ${w}_{1}$ and ${w}_{2}$ in Fig. 1c, were obtained by training the composite model on the given training set.

### Classifiers Based on Existing Methods

The AUC for the TRS was computed by assigning all patients with a TRS of 1 or 2 a value of 1, all patients with a TRS of 3 or 4 a value of 2, and all patients with a TRS greater than 4 a value of 3; i.e., we use the standard TIMI-risk group classification. The HRs were computed by assigning all patients with a TRS >4 to the high-risk group, and all patients with TRS ≤4 to the low-risk group. The two-category NRI was calculated by assigning patients with a TRS > 4 to the event class and patients with TRS <4 to the non-event class. The two category NRI was calculated as^27^:

1 $$NRI=P(up|event)-P(up|nonevent)+P(down|nonevent)-P(down|event)$$

Here, *P* represents probability, *up* denotes patients who were reclassified from the non-event class to the event class, and *down* denotes patients who were reclassified from the event class to the non-event class.

The logistic regression models based on MV, HRV, and DC were constructed by adding to the LR_Hx_ model a single feature corresponding to the MV values for LR_Hx+MV_, HRV values for LR_Hx+HRV_, and DC values for LR_Hx+DC_ for each patient. We note that for HRV we use the HRV-LF/HF metric (which corresponds to the ratio of the low frequency to high frequency power in the heart rate time series), because this metric had the greatest predictive power relative to other HRV metrics in our earlier study of patients post-NSTE-ACS^17^. Details on how MV, HRV, and DC values were calculated using Cohort-1 were described in our previous work^18^.

### Statistical Analysis

Metrics reported on the Cohort-1 dataset were obtained using a bootstrapping procedure in which the entire Cohort-1 dataset was partitioned into a training set consisting of 80% of the data and a testing set consisting of 20% of the data 1,000 times. Reported AUCs are the means of the AUCs on these 1,000 bootstrap rounds. 95% confidence intervals were generated by calculating the standard error of the AUC for each bootstrap split, multiplying the result by 1.96, and adding or subtracting this value from the AUC; the Hanley-McNeil model was used to calculate the standard error^41^. HRs were computed using a Cox proportional hazards model^42^. Reported HRs are the means across bootstrap rounds, and upper and lower 95% confidence intervals are the means of the confidence intervals generated by the Cox model across bootstrap rounds. For the univariate HRs at 60 days, 30 days, and 14 days, as well as for the one-year multivariate HRs, it was necessary to eliminate bootstrap rounds that contained an unsuitably small number of positive examples with which to train and test the models. No more than 50 of the 1000 bootstrap rounds were eliminated for any given model; since maximum number eliminated corresponds to less than 5% of the bootstrap rounds, the results were not expected to be affected significantly. Confidence intervals were generated by adding or subtracting 1.96 times the standard error from the coefficients generated by the Cox model, and HRs and confidence intervals were calculated by exponentiation of the resulting coefficients. The category-free NRI was calculated using equation (1) above, with the change that *up* denotes patients whose raw model outputs increased and *down* denotes patients whose raw model outputs have decreased. The 95% confidence interval for the NRI was calculated by computing the standard error across all bootstrap splits, multiplying the result by 1.96, then adding or subtracting this value from the mean NRI. Statistical significance testing was done using two-sided, paired-sample *t*-tests between each pair of models over the 1,000 bootstrap splits.

All data preprocessing, logistic regression models, and statistical analyses were implemented using the commercial software MATLAB 8.6 (2015b) (The MathWorks, Natick, MA). The RNN was implemented using the Keras software package (F. Chollet, GitHub, 2015).

### Data availability

The clinical data used for these analyses were obtained from multicenter clinical trials where all trial protocols and guidelines for their analyses were approved by the local or central institutional review board at participating centers. These patient datasets are therefore not generally available without permission. However, the trained models (with detailed instructions for their use) and performance metrics are available from the authors upon request.

## Electronic supplementary material

Supplementary Information

## References

[1] Amsterdam EA (2014). AHA/ACC Guideline for the Management of Patients with Non-ST-Elevation Acute Coronary Syndromes: a report of the American College of Cardiology/American Heart Association Task Force on Practice Guidelines. J. Am. Coll. Cardiol..

[2] Mozaffarian D (2016). Heart Disease and Stroke Statistics-2016 Update A Report From the American Heart Association. Circulation.

[3] Cannon CP (2001). Comparison of early invasive and conservative strategies in patients with unstable coronary syndromes treated with the glycoprotein IIb/IIIa inhibitor tirofiban. New Engl. J. Med..

[4] Diderholm E (2002). ST depression in ECG at entry indicates severe coronary lesions and large benefits of an early invasive treatment strategy in unstable coronary artery disease; the FRISC II ECG substudy. The Fast Revascularisation during InStability in Coronary artery disease. Eur Heart J.

[5] Roffi M (2016). 2015 ESC Guidelines for the management of acute coronary syndromes in patients presenting without persistent ST-segment elevation: Task Force for the Management of Acute Coronary Syndromes in Patients Presenting without Persistent ST-Segment Elevation of the European Society of Cardiology (ESC). Eur Heart J.

[6] Bavry AA, Kumbhani DJ, Rassi AN, Bhatt DL, Askari AT (2006). Benefit of Early Invasive Therapy in Acute Coronary Syndromes: A Meta-Analysis of Contemporary Randomized Clinical Trials. J. Am. Coll. Cardiol..

[7] Antman EM (2000). The TIMI risk score for unstable angina/non-ST elevation MI: A method for prognostication and therapeutic decision making. JAMA-J. Am. Med. Assoc..

[8] Morrow DA (2001). Application of the TIMI risk score for ST-Elevation MI in the National Registry of Myocardial Infarction 3. JAMA-J. Am. Med. Assoc..

[9] Granger CB (2003). Predictors of hospital mortality in the global registry of acute coronary events. Arch. Intern. Med..

[10] Savonitto S (1999). Prognostic value of the admission electrocardiogram in acute coronary syndromes. JAMA-J. Am. Med. Assoc..

[11] Cannon CP (1997). The electrocardiogram predicts one-year outcome of patients with unstable angina and non-Q wave myocardial infarction: results of the TIMI III Registry ECG Ancillary Study. Thrombolysis in Myocardial Ischemia. J. Am. Coll. Cardiol..

[12] Kaul P (2001). Prognostic value of ST segment depression in acute coronary syndromes: insights from PARAGON-A applied to GUSTO-IIb. PARAGON-A and GUSTO IIb Investigators. Platelet IIb/IIIa Antagonism for the Reduction of Acute Global Organization Network. J. Am. Coll. Cardiol..

[13] Damman P (2012). Usefulness of the admission electrocardiogram to predict long-term outcomes after non-ST-elevation acute coronary syndrome (from the FRISC II, ICTUS, and RITA-3 [FIR] Trials). Am. J. Cardiol..

[14] Liu, Y. *et al*. ECG Morphological Variability in Beat Space for Risk Stratification After Acute Coronary Syndrome. *JAMA-J*. *Am*. *Med*. *Assoc*. **3**, 10.1161/JAHA.114.000981 (2014). 10.1161/JAHA.114.000981 PMC4309066 24963105

[15] de Araujo Goncalves P, Ferreira J, Aguiar C, Seabra-Gomes R (2005). TIMI, PURSUIT, and GRACE risk scores: sustained prognostic value and interaction with revascularization in NSTE-ACS. Eur Heart J.

[16] Hathaway WR (1998). Prognostic significance of the initial electrocardiogram in patients with acute myocardial infarction. GUSTO-I Investigators. Global Utilization of Streptokinase and t-PA for Occluded Coronary Arteries. JAMA-J. Am. Med. Assoc..

[17] Syed Z (2009). Relation of Death Within 90 Days of Non-ST-Elevation Acute Coronary Syndromes to Variability in Electrocardiographic Morphology. Am. J. Cardiol..

[18] Syed Z, Stultz CM, Scirica BM, Guttag JV (2011). Computationally generated cardiac biomarkers for risk stratification after acute coronary syndrome. Sci. Transl. Med..

[19] Morrow DA (2007). Effects of ranolazine on recurrent cardiovascular events in patients with non-ST-elevation acute coronary syndromes - The MERLIN-TIMI 36 randomized trial. JAMA-J. Am. Med. Assoc..

[20] Morrow DA (2006). Evaluation of a novel anti-ischemic agent in acute coronary syndromes: Design and rationale for the Metabolic Efficiency with Ranolazine for Less Ischemia in Non-ST-elevation acute coronary syndromes (MERLIN)-TIMI 36 trial. Am. Heart J..

[21] Cannon CP (2007). Safety, tolerability, and initial efficacy of AZD6140, the first reversivle oral adenosine diphosphate receptor antagonist, compared with clopidigrel, in patients with non-ST-segment elevation acute coronary syndrome - Primary results of the DISPERSE-2 trial. J. Am. Coll. Cardiol..

[22] Amon M, Jager F (2016). Electrocardiogram ST-Segment Morphology Delineation Method Using Orthogonal Transformations. Plos One.

[23] Erdogan A (2008). Prognostic value of heart rate variability after acute myocardial infarction in the era of immediate reperfusion. Herzschrittmacherther Elektrophysiol.

[24] Bauer A (2006). Deceleration capacity of heart rate as a predictor of mortality after myocardial infarction: cohort study. Lancet.

[25] Tsoi AC, Back A (1997). Discrete time recurrent neural network architectures: A unifying review. Neurocomputing.

[26] LeCun Y, Bengio Y, Hinton G (2015). Deep learning. Nature.

[27] Pencina MJ, D’Agostino RB, Steyerberg EW (2011). Extensions of net reclassification improvement calculations to measure usefulness of new biomarkers. Stat Med.

[28] Nishimura, R. A. *et al*. Prognostic value of predischarge 2-dimensional echocardiogram after acute myocardial infarction. *Am*. *J*. *Cardiol*. **53**, 429–432, 10.1016/0002-9149(84)90007-9. 10.1016/0002-9149(84)90007-9 6695770

[29] Møller, J. E. *et al*. Wall motion score index and ejection fraction for risk stratification after acute myocardial infarction. *Am*. *Heart J*. **151**, 419-425, 10.1016/j.ahj.2005.03.042. 10.1016/j.ahj.2005.03.042 16442909

[30] de Lemos JA (2001). The Prognostic Value of B-Type Natriuretic Peptide in Patients with Acute Coronary Syndromes. New Engl. J. Med..

[31] Richards AM (2003). B-Type Natriuretic Peptides and Ejection Fraction for Prognosis After Myocardial Infarction. Circulation.

[32] Haraldsson H, Edenbrandt L, Ohlsson M (2004). Detecting acute myocardial infarction in the 12-lead ECG using Hermite expansions and neural networks. Artif Intell Med.

[33] Bigi R (2005). Artificial neural networks and robust Bayesian classifiers for risk stratification following uncomplicated myocardial infarction. Int J Cardiol.

[34] Ennis M, Hinton G, Naylor D, Revow M, Tibshirani R (1998). A comparison of statistical learning methods on the GUSTO database. Stat Med.

[35] Harrison RF, Kennedy RL (2005). Artificial neural network models for prediction of acute coronary syndromes using clinical data from the time of presentation. Ann Emerg Med.

[36] Zhao BD, Lu HZ, Chen SF, Liu JL, Wu DY (2017). Convolutional neural networks for time series classification. Journal of Systems Engineering and Electronics.

[37] Heart rate variability. Standards of measurement, physiological interpretation, and clinical use. Task Force of the European Society of Cardiology and the North American Society of Pacing and Electrophysiology. *Eur Heart J* **17**, 354-381 (1996). 8737210

[38] Martinez JP, Almeida R, Olmos S, Rocha AP, Laguna P (2004). A wavelet-based ECG delineator: Evaluation on standard databases. Ieee T Bio-Med Eng.

[39] Camm AJ (1996). Heart rate variability. Standards of measurement, physiological interpretation, and clinical use. Eur Heart J.

[40] Li Q, Mark RG, Clifford GD (2008). Robust heart rate estimation from multiple asynchronous noisy sources using signal quality indices and a Kalman filter. Physiol Meas.

[41] Hanley JA, Mcneil BJ (1982). The Meaning and Use of the Area under a Receiver Operating Characteristic (Roc) Curve. Radiology.

[42] Cox DRRM (1972). and Life-Tables. J R Stat Soc B.

