# Supplementary material for: Machine Learning Improves Risk Stratification After Acute Coronary Syndrome
Source: Sci Rep. 2017 Oct 4;7:12692. doi: 10.1038/s41598-017-12951-x (PMC5627253; doi:10.1038/s41598-017-12951-x)
Supplement: Supplementary file 1 — Supplementary Information [file 41598_2017_12951_MOESM1_ESM.pdf]

Supplementary Materials for:

Machine Learning Improves Risk Stratification After Acute Coronary Syndrome

Paul D. Myers, Benjamin M. Scirica, Collin M. Stultz

Supplementary Materials and Methods

ST-Segment Morphology Delineation

The following description is based on that given in [19]. The use of orthogonal basis functions to represent the morphology of the ST-segment is based on the observation that the functional forms of appropriately chosen basis functions approximate the main categories of ST-segment morphology; these categories are the level with respect to the isoelectric line, slope, and curvature of the ST-segment. Mathematically, the first category corresponds to a constant function, the second to a linear function, and the third to non-linear functions. The set of Legendre polynomials is known to possess such functional forms; the first five Legendre polynomials are:

$$P_0 = 1$$
$$P_1 = x$$
$$P_2 = \frac{1}{2}\left(3x^2 - 1\right)$$
$$P_3 = \frac{1}{2}\left(5x^3 - 3x\right)$$
$$P_4 = \frac{1}{8}\left(35x^4 - 30x^2 + 3\right)$$

Thus, P_0 corresponds to the ST-segment level, P_1 to the slope, and P_2 through P_4 to higher-order curvature. These polynomials may be weighted by coefficients and superimposed to generate an ST-segment, as follows:

$$S(x) = \sum_{i=0}^{4} c_i P_i(x)$$

Here, the c's are the weighting coefficients, the P's are the polynomials defined above, S is the value of the normalised ST-segment voltage, and x is the sample input. The Legendre polynomials may be generated by applying the Gram-Schmidt orthonormalisation procedure to the following matrix:

$$\Omega_{ij} = \left(2\frac{i-1}{M-1} - 1\right)^{j-1}, i = 1,2,...M; j = 1,2,...,M$$

Here, M is the number of sample points in the range [-1,1], and is chosen based on the sampling rate of the ECG signal. In [19], 32 samples were used for a sampling rate of 250 Hz. In the present work, the sampling rate was 128 Hz, necessitating the use of a smaller number of samples; 16 samples were used. Applying the Gram-Schmidt orthonormalisation procedure to $\mathbf{\Omega}$ generates a new matrix $\mathbf{\Phi_L}$, which may be used to transform a vector of ST-segment samples into its constituent Legendre polynomials basis functions. To do so, the ECG signal for a given patient is

partitioned into beats, and each beat is segmented into its constituent waves. The ST-segment of each beat is taken to be all sample points falling between the S-wave and T-wave labels generated by the segmentation algorithm; beats missing either an S-wave or T-wave label or both were rejected as being too noisy to use. The length of the ST-segment can vary widely between beats depending on the variation of the heart rate and condition of the patient over time; segment lengths were found to range between fewer than 16 to more than 32. Segments with fewer than 16 and more than 32 samples were rejected as either being too noisy to use or improperly labeled by the segmentation algorithm. For segments with lengths between 16 and 32 samples, the midpoint of the segment was found and eight samples were taken on either side of it. Doing so helped to ensure that the samples used were part of the true ST-segment, even in the presence of small errors in the placement of the S-wave and T-wave labels. The set of 16 samples is stored in a vector called $\boldsymbol{x}$, which is used to generate the Legendre polynomial representation of the segment as follows:

$$\boldsymbol{s}_j = \frac{\boldsymbol{\Phi}_L^T \boldsymbol{x}_j}{\sigma\left(\boldsymbol{\Phi}_L \boldsymbol{x}_j\right)}$$

Here, $\boldsymbol{s}_j$ is the Legendre polynomial expansion for beat j, $\boldsymbol{x}_j$ is the sample vector for beat j, $\boldsymbol{\Phi}_L$ is as defined above, and σ denotes the standard deviation; T indicates the matrix transpose operation.

Background on Neural Networks

Neural networks consist of a set of interconnected processing units that are loosely inspired by the supposed organisation of the human brain[1]. An example of a processing unit, which is referred to as an artificial neuron in analogy with biological neurons, is shown in Supplementary Fig. S1[2]. The neuron receives inputs from an arbitrary number of other neurons, and each input is multiplied by a weight, which represents the synapse between two neurons. The weighted inputs are then summed in a process called integration, and the resulting sum is given as input to a nonlinear function called an activation function, the output of which may then be sent to other neurons or used in other computations. The choice of activation function depends on the purpose for which the neuron is being used. Common choices include the hyperbolic tangent and sigmoid functions, since these functions are bounded and therefore help prevent computations from becoming unbounded in large networks. A feedforward neural network (FNN), which is shown in Supplementary Fig. S2, is constructed by arranging a number of these neurons into cascaded layers; the name “feedforward” indicates that information flows from the input of the network to the output, without any of the intermediate computations being stored for future use. Each layer of the network consists of an arbitrary number of neurons that receive inputs from the neurons in the preceding layer, if there is one. Each neuron may receive inputs from all or a subset of the neurons in the preceding layer; the term “fully-connected” is applied to layers in which the neurons receive inputs from all of the neurons in the preceding layer. The first layer of the network is called the input layer, and contains as many neurons as there are input variables. The final layer of the network is called the output layer, and contains as many neurons as there are output variables. All intermediate layers, if there are any, are called hidden layers and may contain any number of neurons. Increasing the number of hidden layers generally increases the ability of the network to identify complex patterns in the data, but also increases the risk of overfitting.

Recurrent neural networks (RNNs) are similar to FNNs in that they consist of cascaded layers of interconnected neurons, but unlike FNNs, RNNs store intermediate results for use in future

computations; the term "recurrent" is used to denote this memory-like capability of RNNs. An example of a RNN is shown in Fig. 1B in the main text. The essential difference between the FNN in Fig. S2 and the RNN in Fig. 1B is that the output of the hidden layer in the RNN in one step of the computation is used as input to the hidden layer in the next step of the computation. Reuse of previous results allows the network to identify long-term dependencies in data that follow a sequence, making RNNs useful for the analysis of text, as well as time series data.

The training of a neural network is done in a supervised fashion, wherein the inputs are applied to the network and the resultant outputs are compared with the expected outputs; the training process seeks to optimise the choice of the weights of the interconnections between neurons so as to minimise the error between the expected and computed output. The error is measured by an objective function that is chosen according to the use of the network, and is sent back through the network in order to adjust the weights; this process is called "backpropagation." The adjustment of the weights is typically done using a method based on gradient descent, in which the gradients of the objective function with respect to the weights are computed using the errors calculated above and minimised. While the weights are updated after each training example is seen, the gradients may be updated after the network sees all examples, after it sees each example, or after seeing batches of examples; the first approach is called batch gradient descent, the second is called stochastic gradient descent, and the third is called mini-batch gradient descent. A single round of training in which all examples in the training set are shown to the network is referred to as an "epoch." The order of the training examples is usually shuffled at the beginning of each epoch, and the network is often trained using many epochs.

RNN Classifier Architecture

The time series nature of the Legendre polynomial coefficient data necessitates the use of a classifier capable of processing sequences of data; as discussed above, a RNN is well suited to this problem. The RNN employed consists an input layer of two units, a hidden layer of 13 units, and an output layer of one unit. Although 16 coefficients were generated from each ST-segment, only the first two were used in the final RNN classifier, since higher-order coefficients were found to capture too much noise in the data to offer accurate predictions. The number of hidden units was chosen so as to ensure that the number of free parameters, represented by the weights, of the model was limited to no more than 10% of the number of patients in the training set; with two input units, 13 hidden units, and one output unit, there are 208 weights, which is less than 10% of the patients in the entire Cohort-1 dataset. The classification problem to be solved by the network is binary, so only one output unit is needed. Training of the network was done using 100 epochs with a batch size of 32 patients, as this configuration was found to produce the maximal AUC. The objective function optimised was the binary cross-entropy loss function, which is commonly used for binary classification problems.

Supplementary Figures

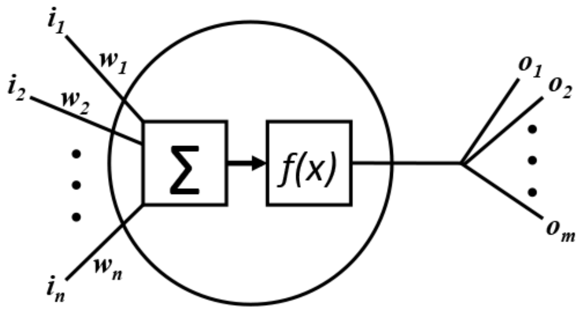

Supplementary Figure S1: Individual processing unit or neuron. Inputs are multiplied by corresponding weights, summed, and passed to a nonlinear activation function. The *i*'s correspond to inputs, the *w*'s to weights, and the *o*'s to outputs[44].

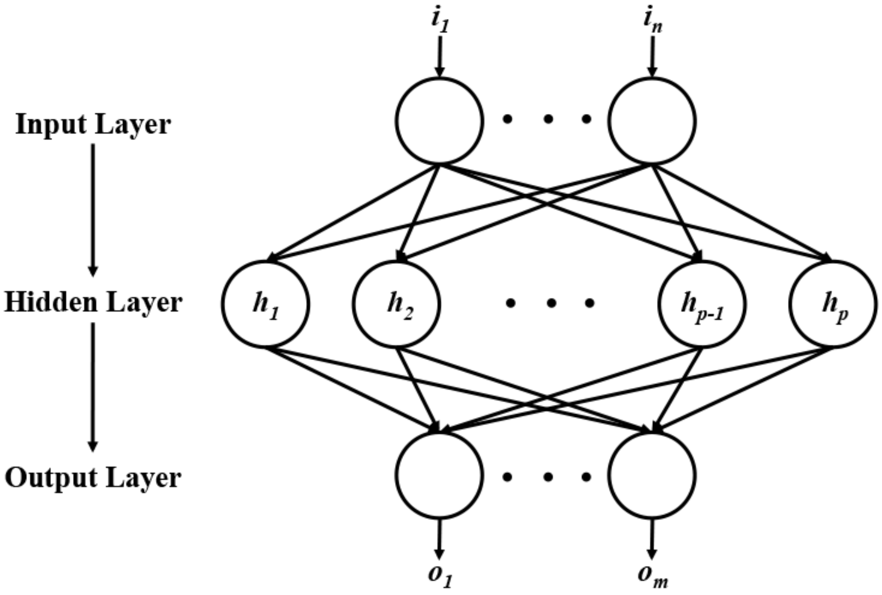

Supplementary Figure S2: Feedforward neural network. Arrows indicate the direction of information flow. The *i*'s correspond to inputs, the *h*'s to hidden units, and the *o*'s to outputs.

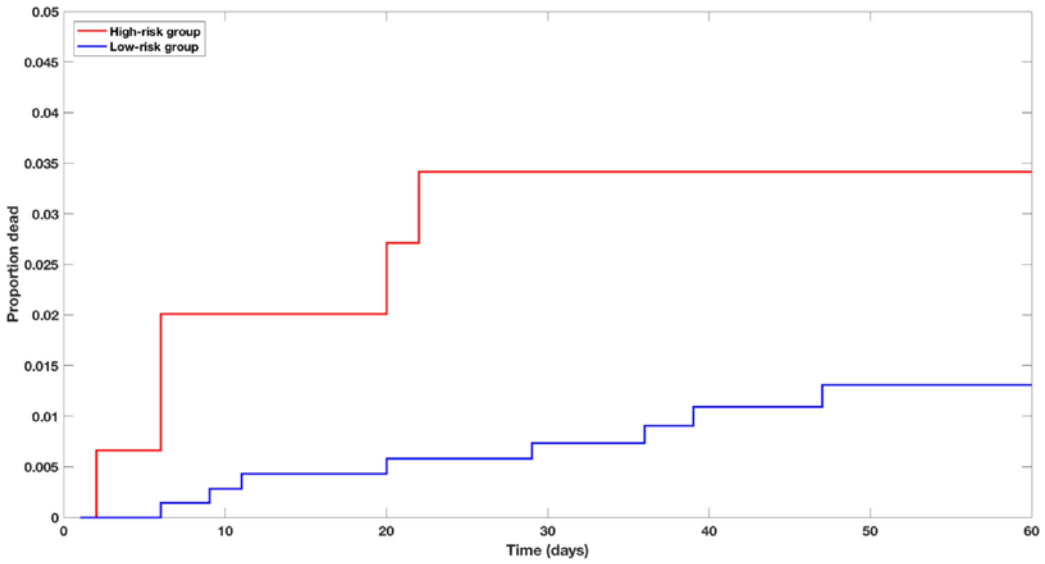

# Patients at Risk

**High-Risk**	150	147	146	145	145	145	145
**Low-Risk**	711	709	708	707	705	704	704

Supplementary Figure S3: Kaplan-Meier survival curves for the LR_{HX+ST} model from Cohort-2. The high-risk group consists of those patients with a raw LR_{HX+ST} prediction value for the Cohort-2 holdout set above the value representing the upper-quartile on the Cohort-1 training set. The number of patients remaining at each labeled time point in each risk group is shown below the plot.

Supplementary Tables

Supplementary Table S1: Univariate hazard ratios for the LR_{Hx}, LR_{ST}, and RNN models from Cohort-1. LR is logistic regression; RNN is recurrent neural network; CI is confidence interval. Hazard Ratios and CIs represent averages over 1000 trials (each trial yields one HR and one 95% CI).

	**Hazard Ratio**	**95% CI**
LR_{Hx}		
1-Year	3.493	1.674 - 7.292
60-Day	4.178	1.378 - 13.020
30-Day	5.117	1.294 - 22.281
14-Day	5.472	1.074 - 31.191
LR_{ST}		
1-Year	3.662	1.752 - 7.657
60-Day	3.098	1.047 - 9.371
30-Day	3.446	0.938 - 13.582
14-Day	3.301	0.693 - 17.536
RNN		
1-Year	3.402	1.631 - 7.098
60-Day	3.419	1.145 - 10.543
30-Day	3.606	0.974 - 14.307
14-Day	3.687	0.766 - 19.784

Supplementary Table S2: Univariate hazard ratios (highest vs. other quartiles) for the comparison models from Cohort-1. CI is confidence interval; TRS if TIMI risk score; LR is logistic regression; MV is morphological variability; HRV is heart rate variability; DC is deceleration capacity. Hazard Ratios and CIs represent averages over 1000 trials (each trial yields one HR and one 95% CI).

	**Hazard Ratio**	**95% CI**
LR_{Hx+MV}		
1-Year	3.960	1.889 - 8.310
60-Day	5.371	1.698 - 17.763
30-Day	5.833	1.444 - 26.156
14-Day	5.709	1.115 - 32.772
LR_{Hx+HRV}		
1-Year	4.126	1.962 - 8.683
60-Day	5.195	1.669 - 16.549
30-Day	6.356	1.538 - 29.242
14-Day	6.524	1.218 - 39.288
LR_{Hx+DC}		
1-Year	3.371	1.617 - 7.029
60-Day	3.609	1.218 - 10.855
30-Day	4.642	1.216 - 19.105
14-Day	5.565	1.087 - 31.946

Supplementary Table S3: Multivariate analysis for the LR_{Hx}, LR_{ST}, and RNN models from Cohort-1. LR is logistic regression; RNN is recurrent neural network; CI is confidence interval; TRS is TIMI risk score; LVEF is left ventricular ejection fraction; BNP is brain natriuretic peptide. HRs and CIs represent averages over 1000 trials (each trial yields one HR and one 95% CI).

	**1-Year HR**	**95% CI**
LR_{Hx}		
TRS	2.551	1.146 - 5.684
TRS+LVEF	1.906	0.698 - 5.237
TRS+BNP	2.174	0.829 - 5.730
TRS+LVEF+BNP	2.083	0.619 - 7.238
LR_{ST}		
TRS	2.966	1.390 - 6.330
TRS+LVEF	2.644	1.010 - 6.971
TRS+BNP	2.791	1.106 - 7.087
TRS+LVEF+BNP	2.918	0.879 - 10.152
RNN		
TRS	2.880	1.364 - 6.081
TRS+LVEF	2.770	1.076 - 7.180
TRS+BNP	2.859	1.148 - 7.165
TRS+LVEF+BNP	3.277	1.000 - 11.239

Supplementary Table S4: Number of CVDs for each subpopulation of Cohort-1. Percentages are with respect to the total patient population.

	**# CVDs**
Age	
> 65	84 (1.91%)
≤ 65	65 (1.48%)
Gender	
Female	52 (1.18%)
Male	97 (2.21%)
Current Smoker	
Yes	31 (0.71%)
No	118 (2.68%)
Hypertension	
Yes	111 (2.53%)
No	38 (0.86%)
Diabetes	
Yes	51 (1.16%)
No	98 (2.23%)
Prior MI	
Yes	46 (1.05%)
No	103 (2.34%)
Prior Angiography	
Yes	55 (1.25%)
No	94 (2.14%)
ST Depression at Enrollment	
Yes	56 (1.27%)
No	93 (2.12%)
TRS	
≤ 4	122 (2.78%)
> 4	27 (0.61%)
LVEF	
> 40%	86 (1.96%)
≤ 40%	10 (0.23%)
BNP	
≤ 80 pg/ml	50 (1.14%)
> 80 pg/ml	44 (1.00%)

References:

1. M. De Beule, E. Maes, O. De Winter, W. Vanlaere, R. Van Impe, Artificial neural networks and risk stratification: A promising combination. *Math. Comput. Model.* **46**, 88-94 (2007).
2. P. A. Jansson, Neural networks: An overview. *Anal. Chem.* **63**, 357A-362A (1991
